# The SMC5/6 complex: An emerging antiviral restriction factor that can silence episomal DNA

**DOI:** 10.1371/journal.ppat.1011180

**Published:** 2023-03-02

**Authors:** Ishak D. Irwan, Bryan R. Cullen

**Affiliations:** Department of Molecular Genetics and Microbiology, Duke University Medical Center, Durham, North Carolina, United States of America; Medical Research Council Laboratory of Molecular Biology, UNITED KINGDOM

Human cells express 3 conformationally similar structural maintenance of chromosome (SMC) complexes called cohesin, condensin, and the SMC5/6 complex [1]. The SMC5/6 complex plays an important role in chromosomal DNA repair, including repair of double-stranded DNA breaks via homologous recombination, and maintenance of replication fork stability. The SMC5/6 complex has also been reported to use ATP hydrolysis to conformationally compact bound DNA. In addition to SMC5 and SMC6, the SMC5/6 complex contains 6 other proteins: nonstructural maintenance of chromosomes element 1 through 4 (NSMCE1-4) and SMC5/6 localization factors 1 and 2 (SLF1 and 2). Of note, NSMCE2 has been shown to be a SUMO E3 ligase and SUMOylation of a range of protein substrates, including the SMC5/6 complex itself, is thought to play a key role in the maintenance of DNA integrity by the SMC5/6 complex [1].

## How does the SMC5/6 complex affect hepatitis B virus gene expression?

Hepatitis B virus (HBV) is a DNA virus with a life cycle that is somewhat similar to retroviruses [2]. While HBV, like retroviruses, encodes a reverse transcriptase that copies the viral DNA genome from a genome-length RNA, HBV differs from retroviruses in that reverse transcription generates DNA genomes from multiple “pre-genomic” RNA transcripts in infected producer cells. A second difference between HBV and retroviruses is that HBV DNA does not normally integrate into the host genome but rather replicates as an episome.

HBV encodes a nonstructural protein, HBx, whose function was for many years enigmatic, though it is essential for viral gene expression and replication [2] as, in the absence of HBx function, HBV episomes are epigenetically silenced [3]. Moreover, nuclear HBx promiscuously activates transcription from extrachromosomal DNA templates, including not only HBV DNA but also transfected expression plasmids, while having no effect on transcription of the same DNA molecule if integrated into a chromosome [4]. Finally, it was known that HBx bound the DNA-damage binding protein 1 (DDB1) subunit of a DDB1-containing E3 ubiquitin ligase [5]. Using a proteomic approach, 2 groups identified the SMC5/6 complex as a target for binding by the HBx-DDB1 E3 ligase complex and showed that this induced the degradation of SMC5 and SMC6 [6,7]. Moreover, the SMC5/6 complex directly bound episomal HBV DNA in the absence of HBx and loss of SMC5/6 complex function rescued the replication of an HBV mutant lacking a functional HBx. Together, these data indicated that the SMC5/6 complex recognizes and binds episomal HBV DNA molecules and then induces their epigenetic silencing. HBx in turn prevents this silencing by inducing the degradation of SMC5 and SMC6. Thus, it is only in the context of infection by an HBx-deficient HBV mutant that the antiviral activity of the SMC5/6 complex is revealed.

## Does the SMC5/6 complex also regulate HIV-1 gene expression?

As noted above, a key difference between HBV and retroviruses such as human immunodeficiency virus type 1 (HIV-1) is that retroviruses include chromosomal integration of the DNA proviral intermediate as an essential step in their replication cycle. In the absence of integrase (IN) function, unintegrated linear HIV-1 DNA generated by reverse transcription of the viral RNA genome is rapidly chromatinized and epigenetically silenced [8] and drugs that inhibit IN function are therefore highly effective antiretrovirals.

The first effort to identify the cellular factors that epigenetically silence extrachromosomal HIV-1 DNA used a limited CRISPR/Cas genetic screen to identify the SMC5/6 complex component SLF2 as critical for this process [9]. These authors then showed that all SMC5/6 complex components, except SLF1, were required for transcriptional silencing of unintegrated HIV-1 DNA. The SMC5/6 complex binds unintegrated HIV-1 DNA and then induces not only the epigenetic silencing but also the compaction of HIV-1 DNA and both effects were proposed to be causally linked to transcriptional repression. Recently, we reported a second CRISPR/Cas genomic screen that also identified the SMC5/6 complex as critical for the epigenetic silencing of unintegrated HIV-1 DNA, though this study differed in that it reported that all 8 components of the SMC5/6 complex, including SLF1, were required for effective silencing [10]. This study also identified the SUMO E3 ligase activity of the SMC5/6 complex component NSMCE2 as essential for silencing. Specifically, a mutant form of NSMCE2 lacking SUMO E3 ligase activity remained able to bind chromatinized, unintegrated HIV-1 DNA, yet had lost the ability to induce both its SUMOylation and its epigenetic silencing. Moreover, a drug that acts as a general inhibitor of protein SUMOylation, called TAK-981, also rescued gene expression from unintegrated HIV-1 proviruses. SUMOylation by the NSMCE2 component of SMC5/6 was required for the initiation but not maintenance of epigenetic silencing, as the ability of TAK-981 to prevent silencing was lost after 24 h post-infection even though TAK-981 retained the ability to deplete SUMO modifications from chromatinized, unintegrated HIV-1 DNA at later times.

A key result reported in this manuscript [10] was the finding that loss of SMC5/6 complex expression or function inhibited the establishment of latent HIV-1 infections in both T cell lines and primary T cells. Latently HIV-1 infected T cells contain an integrated HIV-1 provirus that is transcriptionally silent and they therefore do not produce any viral proteins [11]. Hence, this infection is not cytopathic nor are these cells detectable by immune surveillance. Because latently infected T cells are long lived and retain the ability to randomly reactivate expression from latent HIV-1 proviruses months or even years after initial infection [11], they represent the key factor preventing the complete clearance of HIV-1 from infected individuals, necessitating the lifelong use of antivirals. While the origin of the transcriptionally quiescent, integrated proviruses found in latently infected T cells had previously remained uncertain, these recent data indicate that at least part of the latent reservoir results from the epigenetic silencing of HIV-1 proviruses by the SMC5/6 complex prior to integration.

Prior to the publication of the 2 manuscripts cited above [9,10] identifying the SMC5/6 complex as essential for the epigenetic silencing of unintegrated HIV-1 DNA, the Goff laboratory reported that the human silencing hub (HUSH) complex, consisting of MPP8, TASOR, and PPHLN1, acting in concert with a DNA-binding protein called NP220, was required for the epigenetic silencing of unintegrated DNA generated upon infection of human cells by the animal retrovirus murine leukemia virus (MLV) [12]. However, none of the HUSH complex subunits, or NP220, were identified as required for unintegrated HIV-1 DNA silencing in the 2 genetic screens cited above [9,10] and genetic ablation of the expression of MPP8, TASOR, PPHLN1, or NP220 did not enhance the transcription of unintegrated HIV-1 DNA [9,13]. We note that, while it is not currently known whether the SMC5/6 complex contributes to the silencing of unintegrated MLV DNA, the HUSH complex has been reported to specifically repress the transcription of integrated HIV-1 DNA [14,15]. Whether the epigenetic silencing of unintegrated HIV-1 and MLV DNA in human cells is indeed mediated by 2 distinct mechanisms clearly needs to be more fully elucidated.

## Does the SMC5/6 complex interact with other human DNA viruses?

Given the ability of the SMC5/6 complex to epigenetically silence extrachromosomal HBV and HIV-1 DNA, the obvious question is whether the SMC5/6 complex is also capable of silencing other episomal viral DNAs. If all DNA episomes are indeed targeted by SMC5/6, then replication competent DNA viruses must have some means of avoiding transcriptional silencing and therefore likely encode a factor(s) that inhibits SMC5/6 function. In the case of Epstein–Barr virus (EBV), the viral tegument protein BNRF1 was reported to induce the ubiquitination and degradation of SMC5/6 complexes [16]. Inhibiting this degradation allowed SMC5/6 to bind episomal EBV DNA, interfere with replication compartment formation, and hence inhibit virion production. Interestingly, SUMOylation by the NSMCE2 subunit of SMC5/6 was shown to be involved in this restriction, though the relevant target(s) remains undiscovered.

Similarly, in Kaposi’s sarcoma herpesvirus (KSHV)-infected cells, the SMC5/6 complex was reported to bind episomal KSHV DNA and inhibit viral replication. KSHV avoids this by using the viral protein RTA to ubiquitinate and degrade SMC5/6 complex components [17]. The SMC5/6 complex has also been reported to inhibit the replication of human papilloma virus (HPV). SMC5/6 binds the E2 protein encoded by HPV-31 and depletion of SMC5/6 complexes increases HPV-31 replication in keratinocytes, though the mechanistic basis for this effect remains unknown [18]. SMC5/6 has also been reported to reduce herpes simplex virus 1 replication by binding to episomal viral DNA [19]. Moreover, the SMC5/6 complex inhibits transgene expression from adeno-associated virus (AAV)-based vectors [20] and, intriguingly, SUMOylation was reported to also restrict transduction by AAV-based vectors, though this was not shown to be caused by the SMC5/6 complex [20]. Finally, it is worth noting that the SMC5/6 complex can also inhibit transcription from transfected expression plasmids [21]. Expression of the HBV HBx protein, which degrades SMC5 and SMC6, or knocking out these proteins, activates gene expression from episomal transfected plasmids but fails to activate the same expression plasmids after chromosomal integration [4,21].

## Do unresolved issues exist with regard to the antiviral function of SMC5/6?

While the important role played by the SMC5/6 complex in maintaining chromosome integrity is well established, the evidence that SMC5/6 can epigenetically silence extrachromosomal viral DNA is more recent. As a result, many questions remain unresolved, including how the SMC5/6 complex recognizes extrachromosomal DNA and how SMC5/6, once bound to that DNA, induces silencing. There are also discrepancies in the field that need to be resolved, including whether SLF1 is required for epigenetic silencing by SMC5/6. Moreover, while we recently reported that SUMOylation of chromatinized extrachromosomal viral DNA is a critical step in the induction of epigenetic silencing [10], and chromatin SUMOylation has previously been implicated in transcriptional repression [22], a recent report argued that, while the NSMCE2 is indeed critical for the epigenetic silencing of transfected DNA, its SUMOylation activity is not [21]. Moreover, it will be interesting to more clearly determine if the SMC5/6 complex is indeed capable of restricting gene expression from a range of human DNA viruses and, if so, how these viruses circumvent this restriction. If all episomal DNA viruses antagonize SMC5/6 complex function by, for example, expressing a factor that degrades 1 or more SMC5/6 complex components, as previously reported for HBV HBx, EBV BNRF1, and KSHV RTA, then this implies that drugs that inhibit this process could represent a novel class of potent antivirals. Given the known role of the SMC5/6 complex in maintaining genome stability, it is tempting to speculate that inactivation of the SMC5/6 complex by proteins encoded by viruses such as HBV, EBV, KSHV, and HPV may also contribute to their known oncogenic potential. Finally, while this short review has focused almost entirely on the antiviral potential of the SMC5/6 complex, we note that cohesin, another SMC complex consisting of SMC1, SMC3, and associated factors, has also been shown to repress early gene expression from KSHV DNA during latency [23,24]. It will therefore be important for future work to more fully address whether other SMC complexes, including not only cohesin but also condensin, also have the potential to inhibit the transcription of viral DNA and might therefore also be targeted for inactivation.
